# Homopositivity Across Europe: Predictors, Pathways, and Moderators

**DOI:** 10.1007/s10508-023-02531-8

**Published:** 2023-01-20

**Authors:** Adam Whitworth, Angelo Moretti

**Affiliations:** 1 Department of Work, Employment and Organisation, Strathclyde Business School, University of Strathclyde, Glasgow, G4 0QU UK; 2 Methodology and Statistics, Utrecht University, Utrecht, Netherlands

**Keywords:** Homopositivity, Homonegativity, Structural equation modelling, Mediation, European Social Survey, Sexual orientation

## Abstract

Although homopositivity, the attitudinal acceptance of homosexuality, has generally increased across Western societies, there remains considerable homonegativity across certain regions of the world as well as certain demographic and socioeconomic groups. Although previous cross-national research has successfully identified the key factors affecting homopositive attitudes, the literature neglects both potentially key mediation pathways and moderating interactions between those factors that may unlock more nuanced understanding of these variations in homopositive attitudes across individuals and places. In response, the present study innovatively applied a multivariate structural equation modelling approach to the latest Wave 9 (2018 data) of the large-scale cross-national European Social Survey data in order to shed new light on these currently neglected predictors, pathways, and moderating influences on homopositive attitudes. It used a three-item latent variable to measure the homopositive attitudes outcomes construct. Its explanatory variables were focused across three key sets of factors identified in theoretical and empirical literature (socioeconomics, religiosity, and values) alongside various wider controls. Our analyses made several innovative methodological and empirical contributions to existing debate. Key innovative findings include the original identification of important indirect effects of religious beliefs on homopositive attitudes via religious practices, important indirect effects of education on homopositive attitudes via household income, and the role of national welfare regimes to homopositive attitudes (and with its effects interestingly not moderated by household income).

## Introduction

Although homopositivity, the attitudinal acceptance of homosexuality, has generally increased across Western societies there remains considerable homonegativity across certain regions of the world including Africa, Eastern Europe, and parts of Asia (Alozie et al., 2017; IGLA Europe, 2019; Roberts, 2019). In addition, there is evidence to suggest that legal and policy protections for homosexuality are far from inevitable, with several eastern European nations showing reversals in previous progress towards homopositivity between 2018 and 2019 (IGLA Europe, 2019). Within nations too, there remains widespread variation in homopositive attitudes across individuals. Several cross-national quantitative studies have examined the reasons for variation in homonegative attitudes across Europe (Gerhards, 2010; Hooghe & Meeusen, 2013; Kuntz et al., 2015; Slenders et al., 2014; Takacs et al., 2016; van den Akker et al., 2013), “Western” nations (Hildebrandt et al., 2017) and globally (Hadler & Symons, 2018; Hildebrandt et al., 2019; Kenny & Patel, 2017; Redman, 2018; Roberts, 2019; Souza & Cribari-Neto, 2015). A range of further studies have posed similar questions of specific regions and individual nations including Taiwan (Cheng et al., 2016; Zhou & Hu, 2020), Korea (Youn, 2018), China (Zhou & Hu, 2020), Singapore (Zhou & Hu, 2020), the USA (Haney, 2016; Worthen et al., 2019), Netherlands (Haney, 2016), Italy (Worthen et al., 2019), Spain (Worthen et al., 2019), Africa (Alozie et al., 2017), and Eastern Europe (Bolzendahl & Gracheva, 2018).

Taken together these studies have successfully identified many of the key predictors of homopositive attitudes including age, religiosity, gender, education, and income as well as the country’s level of economic development and general level of religiosity. Analysing recently released survey data for 36,015 individuals across 19 European nations, the present article takes as its basic starting point a similar multiple regression modelling strategy to previous studies including these and further explanatory factors as a reflection of the current state of the art in cross-national understanding of homopositive attitudes. From this basic starting point, the paper advances in a series of ways to make three main contributions to the existing literature.

Firstly, while previous cross-national research has successfully identified the key factors affecting homopositive attitudes, current literature neglects possible mediation pathways between those explanatory factors. For the first time in the literature, the present paper embraces the flexibility around path analysis within a multivariate structural equation modelling (SEM) framework to bring the exploration of key mediation pathways to the heart of the analysis. The exploration of such mediation pathways is important to understand the indirect as well as direct effects of key explanatory variables as well as to shine a light on the processes at play in shaping homopositive attitudes.

Secondly, previous research pays relatively little attention to the potential moderation of key explanatory factors, with some notable exceptions (Hildebrand et al., 2019; Kuntz et al., 2015; Worthen et al., 2017). As such, the current evidence base for the most part assumes that the effects found on key explanatory variables such as religiosity, income, and values simply apply equally, which may not be the case empirically. In contrast, the present study examines the potential for important interaction effects. Specifically, novel within our analyses is the examination of how the effect of religious beliefs may vary by religious denomination—two key and related factors not previously examined in terms of their potential moderation. Also novel is our attention to how the effects of household income might vary by national welfare regime—a factor found in various strands of welfare scholarship to be important to explaining cross-national differences in a range of cultures and outcomes (Esping-Andersen, 1990; Pierson, 2000; Powell et al., 2020), but that is largely neglected in previous scholarship into homopositivity.

Thirdly, although the idea of homopositivity is widely understood in general terms its practical measurement as an outcome indicator lacks attention or debate in the literature, despite the clear importance of its specification to findings. Previous studies select one outcome indicator to capture the concept, often (though by no means always) a general question asking to what extent respondents agree with the statement that gay and lesbian individuals should be free to live their life as they wish. Instead, the present study acknowledges that homopositivity should instead be treated as a latent concept with multiple dimensions and whose nature and strength of feeling may vary dependent upon the specific dimension in question (Preuss et al., 2020). To enable this, our analyses measure homopositivity as a latent outcome comprised of three conceptually distinct indicators of support for homosexuality.

The existing body of theory and evidence highlights four main sets of factors key to shaping homopositive attitudes, even if individual studies frequently do not incorporate all four: religiosity; socioeconomics; basic human values; and country-level contexts. These blocks of explanatory factors form the basis of the present article’s theoretical and empirical structure and contributions.

### Religiosity

Religiosity is consistently found to be a major determinant of homonegativity, with the strength of religious belief, the degree of regular participation in religious practices, and religious denomination each playing a role (Gerhards, 2010; Herek, 2002; Hildebrandt et al., 2019; Hooghe & Meeusen, 2013; Kuntz et al., 2015; Slenders et al., 2014; van den Akker et al., 2013). As would be expected, the greater the level of religiosity the less inclined individuals tend to be towards homopositivity, in terms of both the strength of religious belief and the frequency of religious practices. Variation can also be seen across religious denominations, with individuals of Muslim faith tending to be somewhat more homonegative than other religious denominations, even after having controlled for levels of religious belief and participation alongside other factors (Hooghe & Meeusen, 2013; Slenders et al., 2014; van den Akker et al., 2013). At a wider societal level, research also finds that the general level of religiosity can affect individual’s attitudes towards homosexuality (Kollman, 2007; Slenders et al., 2014). This may be due to religious institutions as anchor institutions of social messaging and education or the increased likelihood of social interactions with people of religious faith (Moore & Vanneman, 2003).

However, despite religious beliefs, practices, and denominations each being found to be important factors in shaping homopositive attitudes, previous studies do not consider the theoretical or empirical possibilities of either mediation pathways or moderation effects between them. Our analyses provide two original contributions for the literature in these regards. Firstly, the modelling explores the potential indirect effect of religious beliefs on homopositive attitudes through its mediation with religious practices. Secondly, our later modelling includes an interaction effect between religious beliefs and religious denomination in order to explore whether there is any evidence that the effects of these religious beliefs vary across different faith groups.

### Socioeconomics

Research consistently finds a positive association between higher levels of education and more tolerant attitudes towards homosexuality (Alozie et al., 2017; Bolzendahl & Gracheva, 2018; Gerhards, 2010; Haney, 2016; Hadler & Symons, 2018; Hooghe & Meeusen, 2013; Kuntz et al., 2015; Redman, 2018; Slenders et al., 2014; Takacs et al., 2016; van den Akker et al., 2013), although the trend towards greater acceptance of homosexuality is visible at all educational levels (Treas, 2002). Equally, although less widely analysed, previous studies also find a positive association between higher levels of household income and more homopositive attitudes (Slenders et al., 2014; Zhou & Hu, 2020). Our SEM models similarly examine the direct effects of both education and household income on homopositive attitudes. However, our analyses go further than current scholarship in also exploring the potential indirect effects of education on homopositivity through household income, a mediation pathway justified by evidence of the financial returns to education and training (Oreopolous & Gunderson, 2020; Psacharopoulos & Patrinos, 2018).

Finally, while household income is found to shape attitudes towards homosexuality (Slenders et al., 2014; Zhou & Hu, 2020), the possible role of the national welfare regime in moderating those links is neglected in previous research, with notable exceptions (Takacs et al., 2016). This is surprising given the demonstration in welfare regimes scholarship of the relevance of welfare regimes to shaping a range of cross-national differences in expectations, behaviours, and outcomes (Esping-Andersen, 1990; Pierson, 2000; Powell et al., 2020). Specific to our analyses, the nature of the national welfare regime has been found to be of relevance to explaining attitudes towards other social cleavages such as immigration (Crepaz & Damron, 2009) and plays an important role in shaping and mediating low income in notably differing ways (Esping-Andersen, 1990; Powell et al., 2020). In later analyses, our modelling advances to explore the potential role of welfare regime both as a direct effect on homopositive attitudes as well as a possible moderator of the effect of household income on homopositive attitudes.

### Basic Human Values

Research into the content, structure, and consequences of basic human values has grown rapidly since Schwartz’s pioneering work around three decades ago (Schwartz, 1992). A recent review article summarises that “values predict a large variety of attitudes, preferences and overt behaviors” (Sagiv et al., 2017, p. 630). In brief, Schwartz’s value theory presents a circle of ten basic human values that cover four main domains of openness to change, self-transcendence, conservation, and self-enhancement that relate to one another in complementarity as well as tension. In doing so, Schwartz’s value theory resonates with Herek’s influential research into homonegative values and attitudes, in particular the call to complement the focus on homophobia with a broader recognition of the constructs and processes of sexual stigma, heterosexism, and sexual prejudice argued to underlie the structures, ideologies, and power relations that perpetuate hostility towards homosexuals (Herek, 2004; Herk & McLemore, 2013).

The relevance of human values to homopositive attitudes has been well demonstrated in the existing research literature (Fitzgerald et al., 2014; Gerhards, 2010; van den Akker et al., 2013; Vicario et al., 2005). Our modelling approach advances these studies by analysing the effects of a more detailed set of values than previous studies as well as by exploring the potential role of a country’s LGBT-friendly legislative arrangements in moderating the effects of those basic human values. In doing so, our analyses unpack potentially hidden heterogeneity in effects and moderations as compared with previous key studies (Kuntz et al., 2015).

### Further Controls

Further controls at both the individual and country level are also included in the modelling. Older individuals and males are, on average, found in previous studies to be less tolerant of homosexuality than younger individuals (Alozie et al., 2017; Fitzgerald et al., 2014; Haney, 2016; Herek, 2002; Hooghe & Meeusen, 2013; Kuntz et al., 2015; Redman, 2018; Slenders et al., 2014; Zhou & Hu, 2020). Three further individual-level control variables—poor health, presence of dependent children, and general life satisfaction—are included. These factors are not included in previous studies, and their potential relevance is therefore unclear. It is hypothesized that having children and being generally more satisfied may relate positively to tolerance for homosexuality because of a desire for one’s child to be accepted unconditionally by others and due to a greater ease with others flowing from greater ease with oneself. At the country leve,l further controls are added relating to the level of economic development (Gerhards, 2010; Kenny & Patel, 2017; Roberts, 2019; Slenders et al., 2014; Souza & Cribari-Neto, 2015) and the general level of religious affiliation (Hadler & Symons, 2018; Kuntz et al., 2015) based on previous findings demonstrating their relevance.

## Method

### Participants

Our empirical work makes use of the latest round (Round 9) of the European Social Survey (ESS) released in late 2019 and relating to data collected during 2018. The ESS has been collected bi-annually since 2001 and has well-established survey sampling, data collection, and weighting procedures as well as detailed documentation (ESS, 2021). The Round 9 survey wave contains data for 36,015 individuals based on strict random probability methods from 19 European countries (Austria, Belgium, Bulgaria, Cyprus, Czech Republic, Estonia, Finland, France, Germany, Hungary, Ireland, Italy, Netherlands, Norway, Poland, Serbia, Slovenia, Switzerland, UK). National samples are representative of the population aged fifteen and over resident within private households, regardless of their nationality, citizenship, or language.

### Measures and Procedure

#### The Basic SEM Modelling Strategy

Although not used in previous in studies in the literature, SEM offers a flexible statistical framework to incorporate our theoretical innovations around the examination of homopositivity as a latent concept, mediation pathways, moderation, and moderated mediation (Edwards & Lambert, 2007; Kline, 2011). All models are conducted using the “sem” command in Stata. The final model employs moderated multiple regression and given the advice to use unstandardized coefficients in such models (Kline, 2011; Whisman & McClelland, 2005); all models are interpreted using unstandardized coefficients throughout for consistency. All analyses are stratified by country and survey weighted as per the ESS guidance (ESS, 2014).

**Fig. 1:**
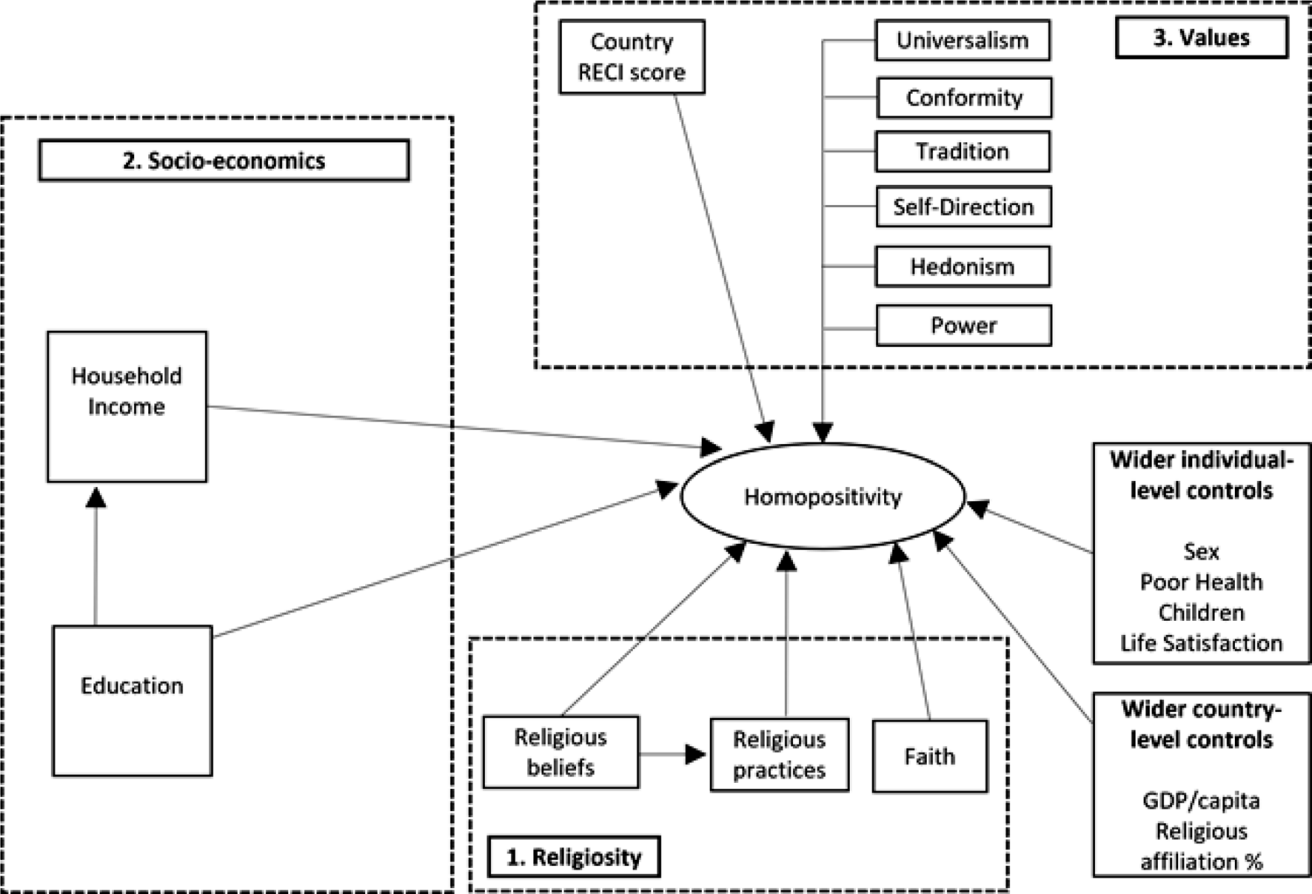
Basic SEM starting point

Figure 1 provides a visual summary of the basic SEM modelling strategy with the selection of variables guided by existing research as outlined above. As is standard in SEM notation, the homopositivity outcome is visualized as an oval to denote its incorporation as a latent factor while variables in rectangles denote single indicators. The modelling strategy is oriented around the three main theoretical blocks of religiosity, socioeconomics, and basic human values, with wider controls also included.

#### The Latent Homopositivity Outcome

As noted above, in contrast to previous research our analyses recognize the potentially multi-dimensional nature of the homopositivity concept and as such measure it via confirmatory factor analysis as a latent factor made up of three indicator variables that capture distinct attitudinal dimensions: “gay and lesbians should be free to live their life as they wish?”; “would you be ashamed if a close family member was gay or lesbian?”; and “gay and lesbian couples should have the right to adopt children?” Each question was recoded 1 to 5 such that higher values relate to more positive attitudes towards homosexuality and goodness of fit statistics support this single factor (RMSEA < 0.06, SRMR < 0.08, TLI ≥ 0.95, CFI ≥ 0.95).

#### Explanatory Variables

Figure 1 summarizes the range of explanatory variables included for each survey respondent. Regards religiosity, literature highlights the relevance of religious beliefs, practices and denomination. Religious beliefs are measured on a 0–10 scale where higher scores relate to stronger religious beliefs. Religious practices are captured by the frequency with which individuals attend routine religious services outside of special occasions such as weddings. This variable is coded 1 to 7 where higher scores relate to greater frequencies of religious practice. Finally, religious denomination is included as dummy variables relating to being of Catholic, Protestant, Eastern Orthodox, Islamic or Other faith, and where No Religion as used as the reference group.

In the socioeconomic theoretical block, explanatory variables relating to education, income and, in later modelling, national welfare regimes.^1^ Education is coded into categories of “low,” “medium,” and “high” with “low” used as the reference category. Household income is classified into deciles with one being the poorest decile and ten being the wealthiest decile. A final model incorporates national welfare systems as categorized into five commonly used regime types (Powell et al., 2020): Eastern European (Bulgaria, Hungary, Poland, Serbia, Slovenia, Czech Republic) as the reference group; Nordic (Finland, Norway, Netherlands); Liberal (Ireland, United Kingdom); Corporatist (Austria, Belgium, France, Germany, Switzerland); and Southern (Spain, Italy, Cyprus).

A final theoretical block relates to basic human values and, in later modelling, its potential moderation by the national legislative and policy framework regards homosexuality. Basic human values of universalism, conformity, tradition, self-direction, hedonism, and power are included in our analyses. Each is coded one to six where higher scores correspond to a greater reporting of that value by the individual. The country’s legal and policy regulation of homosexuality is incorporated as the 2019 Rainbow Europe Country Index (RECI) score (IGLA Europe, 2019). The scores are a composite index of 46 criteria across the six domains of equality and non-discrimination, family, hate crime and hate speech, legal gender recognition and bodily integrity, civil society space, and asylum. National scores range from zero to one hundred where higher scores denote greater support for homosexuality.

A range of wider relevant controls are included at the individual and country level. At the individual level, sex is included as a binary variable with male as the reference category. Health condition is included as a binary variable with good health as the reference category and poor health as the dummy.^2^ The presence of dependent children is included as a binary variable with no dependent children as the reference category and having dependent children as the dummy. Life satisfaction is included as a binary variable with poor life satisfaction as the reference category and high life satisfaction as the dummy.^3^ At the country level, GDP per capita (expressed in 2018 US dollars) and the percentage of the population expressing a religious affiliation in 2018 are included. GDP data are sourced from the World Bank (World Bank, 2020) and national religious affiliation data from the Pew Research Center (Pew Global Research Center, 2018, 2020).

## Results

### Understanding Homopositive Attitudes Across Europe

**Fig. 2:**
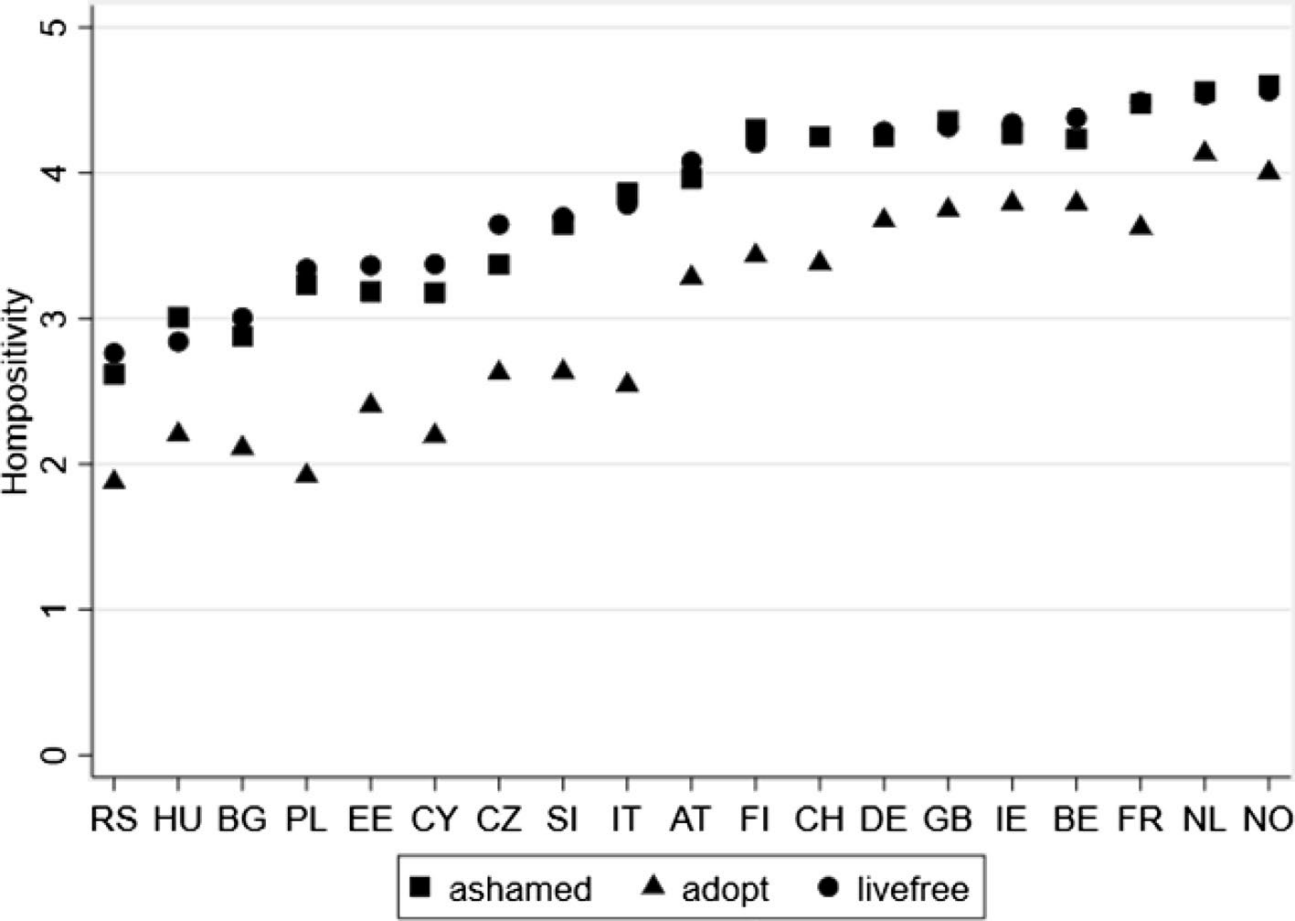
Variation in attitudes towards homosexuality across and within European nations

To provide some baseline context for the analyses, Fig. 2 shows national survey means for all three indicators of attitudes of homosexuality within our latent outcome measure. All three indicators are measured on a five-point scale where higher values relate to greater tolerance of homosexuality. Acceptance of homosexuality varies widely across Europe from widespread acceptance across Western European and Scandinavian nations to relatively widespread disapproval across Eastern Europe. Variation in support for homosexuality is also evident, with the rights of homosexuals to adopt and raise children seeming more challenging for individuals to accept compared to the rights of homosexuals to live freely with their sexuality or whether a sibling’s homosexuality would be a source of shame.

### Initial SEM Modelling

**Fig. 3:**
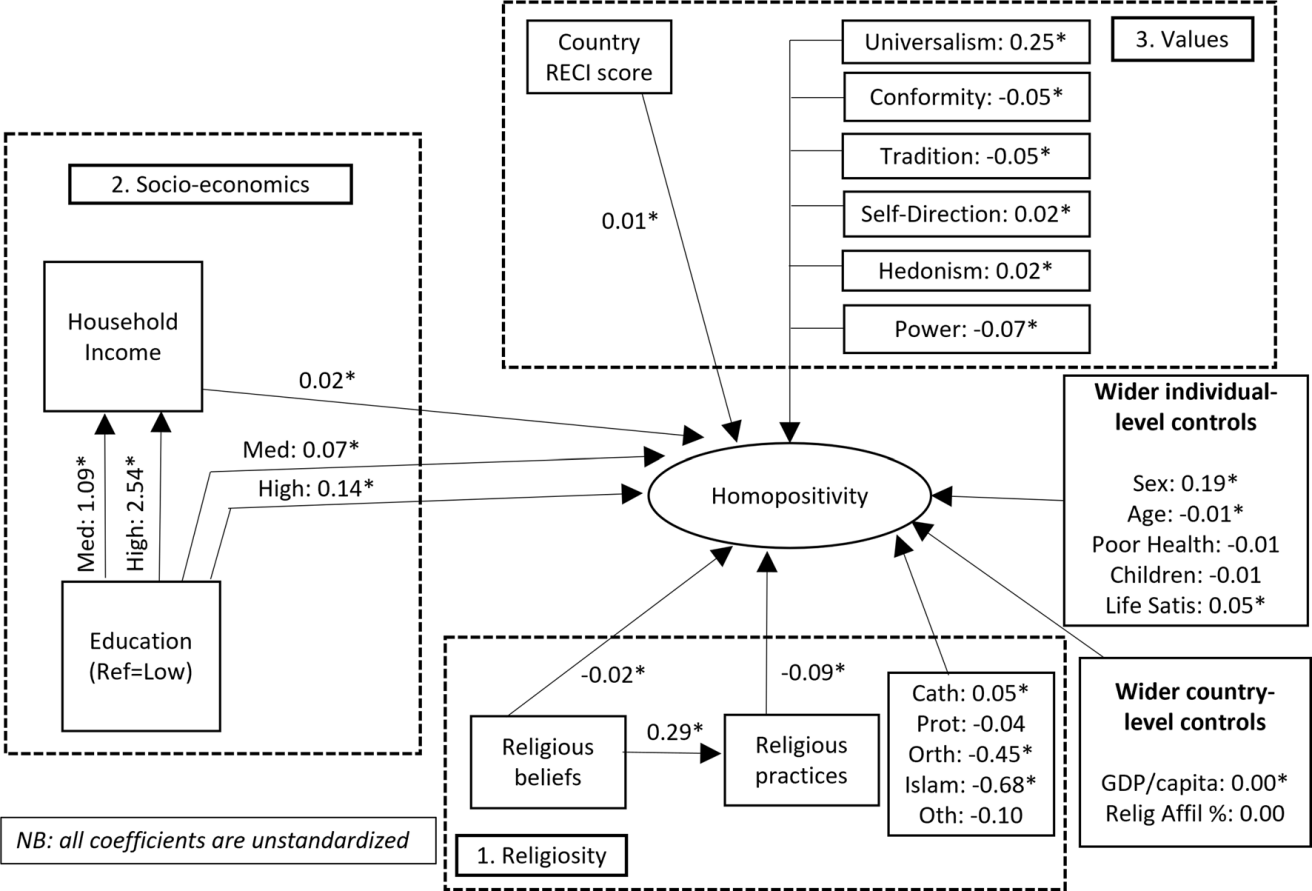
Initial SEM model of homopositive attitudes in Europe

Figure 3 moves on to show the results of our initial SEM model of homopositive attitudes in Europe. All coefficients are unstandardized and can therefore be interpreted as the expected change in homopositive attitudes along its five-point scale (where higher values denote more positive attitudes towards homosexuality) for every unit change in the explanatory variable. Asterisks denote effects that are statistically significant at the 5% level. It will be noted that the individual-level explanatory factors benefit from a large sample size from which to detect even modest effect sizes with statistical significance while the country-level explanatory factors rely on a far smaller analytic sample size with which to detect the statistical significance of those country-level effects. The overall model fit is good with an *R*-squared of 69%.

In the religiosity block, Fig. 3 highlights that religious attitudes, practices, and denomination all play statistically significant roles in accounting for variation in homopositive attitudes across survey respondents. Other things equal, each point increase in religious beliefs associates with a 0.02 decrease in homopositive attitudes while each point increase in religious practices scale is expected to associate with a 0.09 point decrease in homopositive attitudes. Thus, across their full scales these factors associate with large total effects in attitudes towards homosexuality. These analyses additionally advance previous research in drawing attention to the potential indirect effect of religious beliefs on homopositivity mediated through religious practices. These are two factors identified as important but modelled without connection in previous research. Figure 3 shows that each point increase in religious beliefs associates on average with a 0.29 point increase in religious practices, other things equal. As such, in addition to their direct effects religious beliefs also have notable indirect effects on homopositive attitudes through their association with religious practices. Finally, some variation is seen across different religious denominations. Most notably, individuals of Eastern Orthodox and Islamic faith on average show less positive attitudes towards homosexuality, other things equal.

Across the socioeconomic factors, the potential effects of education and household income are summarized in Fig. 3. There are two notable differences to previous research. Firstly, while education and income have both been identified as important to homopositivity in previous research they have been treated as unconnected factors. In contrast, our modelling strategy examines the potential for additional indirect effects of education on homopositivity mediated through household income. Other things equal, having either medium or high level education associates positively with homopositive attitudes compared to the reference category of low education, although these effects are modest in magnitude. Other things equal, for each decile wealthier that a household is there is an expected direct increase of 0.02 points on the homopositivity outcome. Education also shows marked indirect effects on homopositive attitudes mediated through household income, which are roughly one third to one half again the size of the direct effects between education and homopositivity.

As for basic human values, each shows statistically significant estimates and runs in its expected direction. Controlling for other factors, universalism shows the strongest association with homopositivity with a one point increase in the universalism value score expected on average to associate with a 0.25 increase in homopositive attitudes. Tradition, self-direction, and hedonism show smaller positive associations with homopositivity, other things equal, while tradition, conformity, and power show smaller negative associations with homopositivity. The country’s RECI score for LGBT-friendly legislative and policy frameworks shows a small but statistically significant relationship with homopositivity.

Of the wider controls, as expected females tend to show slightly higher scores than males and homopositive attitudes tend to decrease somewhat with every extra year in age, other things equal. None of the wider controls show significant and marked effects.

### Advancing the SEM Modelling: Exploring Moderation and Moderated Mediation

**Fig. 4:**
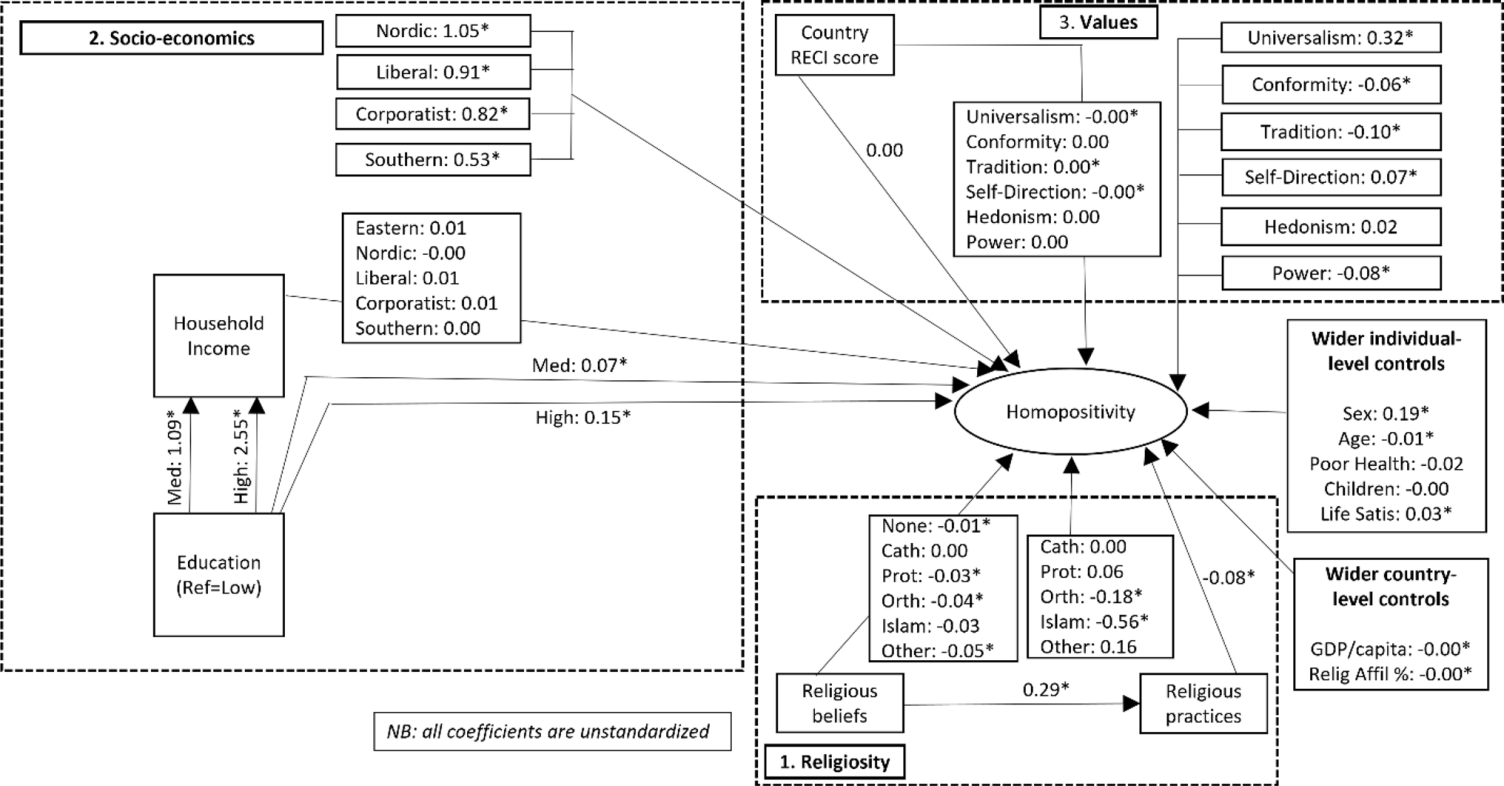
Final SEM model of homopositive attitudes in Europe

Stepping back and reflecting on the SEM modelling above highlights the original insights that are able to be gained through its incorporation of key mediation pathways and latent concepts when compared to the standard regression modelling techniques that dominate the current literature. Figure 4 advances these analyses further by the addition of potentially important yet currently neglected moderation effects and moderated mediation pathways across each of the three core theoretical blocks. Moderated mediation refers to a mediated effect that varies across the levels of a moderator variable (Edwards & Lambert, 2007:6). In the analyses presented in Fig. 4, for example, we allow the mediation pathway between household income and homopositivity to be moderated by welfare regime. In doing so the modelling offers a level of detail and sophistication in theoretical explorations not seen within current scholarship in the field. The overall model fit is good with a slightly increased *R*-squared value of 72%.

Looking first at the religiosity block, included now in Fig. 4 is an interaction between religious beliefs and religious denomination in order to examine for the first time in the literature whether there is any evidence of varying effects of religious belief across faith group. To the centre-right are the main effects of religious denomination flowing directly into the homopositive attitudes outcome. Unlike Fig. 3, given the presence of the interaction terms these main effects relate now only to the situation where religious beliefs take the value zero (which are reported in the data by individuals across all religious denominations, perhaps surprisingly) on its 0–10 scale. At this low level of religious belief individuals with Catholic, Protestant, or Other faiths show no significant difference in homopositive attitudes compared to the reference category of individuals with no denomination. In contrast, individuals of Eastern Orthodox and especially Islamic faith show notably less positive attitudes towards homosexuality, other things equal. These main effects can be considered a starting point in this more subtle understanding of the relationship between the strength of religious beliefs and homopositive attitudes across different faith groups. The path between religious beliefs and homopositive attitudes to the left then shows how the effects of religious belief vary for each religious denomination as the strength of religious belief increases. It will be remembered that the reference category for the religious denomination variable is those with no religious denomination. Other things equal, for these individuals with no stated denomination each point increase on the religious beliefs scale associates with a small but statistically significant decrease in homopositive attitudes. Individuals of Catholic and Islamic faith are suggested to share that same effect while individuals of Protestant, Eastern Orthodox, and Other denominations show somewhat stronger negative associations between increased religious beliefs and homopositive attitudes, other things equal. Finally, religious beliefs continue to show the same positive association with religious practices and those religious practices in turn continue to show the same negative association with homopositive attitudes, other things equal.

Regards socioeconomic factors, new to Fig. 4 is the exploration of whether the positive association between household income and homopositive attitudes found in Fig. 3 shows variation across different welfare regimes. Given the presence of this interaction effect between household income and welfare regime, the welfare regime main effects in the top section of the socioeconomic block in Fig. 4 gives the expected effects of welfare regime for the poorest household income decile only. At this lowest decile of household income, all welfare regimes show large and statistically significant positive associations with homopositive attitudes compared with their Eastern European regime reference group, controlling for other factors. As expected, the Nordic regime shows the largest effect size with a whole point increase in the homopositivity attitudes score expected compared to the reference Eastern welfare regime, other things equal, with Liberal and Corporatist showing slightly smaller but still meaningful effect sizes. The more traditional familialistic and Catholic Southern European welfare regime shows at 0.53 a positive effect around half the size of that seen in the Nordic regime. The welfare regime effects shown on the pathway between household income and homopositive attitudes then reflect the potentially varying effects of household income on homopositive attitudes in each welfare regime type. There is no evidence of varying household income effects across welfare regimes. Taken together, these findings suggest that welfare regime does matter to the shaping of homopositive attitudes but, and perhaps contrary to initial expectations, that it does so less as a moderator of material financial pathways between lower income and homopositive attitudes and instead more as a reflection of their differing cultural and ideological regimes.

For basic human values, Fig. 4 progresses to explore whether the effects of those values seem to be moderated by the country’s legal and policy framework around homosexuality as captured by its RECI score. To the far right are shown the main effects of these basic human values when RECI scores are equal to zero. All but one of these values are statistically significant and effect sizes remain in line with those found previously without the interaction term. There remains limited evidence for the importance of national legislative and policy frameworks on homopositve outcomes, with the direct effect of RECI on homopositive outcomes being statistically insignificant and with a zero effect size. There is also not strong evidence of any variation in the effects of basic human values on homopositivity according to the national framework of LGBT support: half of the human values do not show statistically significant effects and none of the values show effect sizes notably different to zero.

## Discussion

A body of cross-national quantitative studies have built up a valuable picture of the key explanatory factors that explain variation in homopositive/homonegative attitudes across individuals and nations. The present study makes several original contributions to that current understanding through its original use of theoretically guided structural equation modelling.

An initial contribution concerns the specification of the homopositivity outcome variable. While current studies select a (frequently different) single indicator to measure its outcome indicator, the present study encourages greater explicit consideration and debate of this key specification. The present study embraces its ability to readily incorporate latent factors into its SEM framework to instead measure the inherently latent concept of homopositivity from three distinct yet equally plausible underlying indicators. Model comparisons show that the main messages from the findings remain broadly consistent across all specifications of the outcome indicator. However, differences in effect sizes and in reduced model power are evident when single indicators as used as the outcome measure.

Religiosity has been found in numerous previous studies to be a key factor in explaining homopositive attitudes in terms of religious beliefs, practices, and faith groups. Our analyses include all three and findings make two main contributions in this area. Firstly, our models allow a mediation pathway between religious beliefs and religious practices for the first time in the literature and this highlights the strong role of religious beliefs in driving religious practices. This illustrates a key process through which beliefs affect homopositive attitudes via their influence on religious behaviours. It also highlights that the total effect of religious beliefs on homopositive attitudes—by which is meant its direct effects plus its indirect effects mediated via religious practices—are notably larger than previous studies have identified via direct effects alone. Secondly, our analyses include detailed interaction terms between religious beliefs and religious denomination in order to explore the potential for varying effects of beliefs across denominations, in contrast to existing scholarship which assumes uniform effects. Our analyses show that although religious beliefs are important to homopositive attitudes in all faith groups their effect size differs across denominations. Specifically, other things equal Eastern Orthodox and especially Islamic faith show notably less positive attitudes towards homosexuality at low levels of religious belief while individuals of Protestant, Eastern Orthodox, and Other denominations show larger expected increases homonegative attitudes as strength of belief increases compared to other faith groups.

In terms of the socioeconomic drivers of homopositive attitudes our findings make three contributions to the literature. Firstly, they newly illustrate that education has marked indirect effects on homopositive attitudes mediated through household income in addition to the direct effects of both education and household income on homopositivity evidenced in previous research. Secondly, our analyses bring new insights into the role of welfare regimes in affecting homopositive attitudes directly as well as in moderating the effects of low income. The Nordic regime shows the largest positive association with homopositive attitudes and the Eastern European regime the least positive association, other things equal, with Liberal, Corporatist, and Southern European regimes falling in between those extremes. Thirdly, our analyses highlight that household income shows a positive association with homopositve outcomes and that this effect does not vary across welfare regimes despite their markedly differing propensities to mitigate financial risks and losses, particularly at lower income levels. Taken together these findings suggest that welfare regimes do matter to the shaping of homopositive attitudes and hence should be included into future research in the field. However, and perhaps contrary to initial expectations, these findings suggest that they do so less as a moderator of material financial and instead as a reflection of their differing cultural and ideological regimes.

Finally, our analyses confirm the relevance of key basic human values to homopositive attitudes, with Universalism showing a particularly strong positive association with homopositive attitudes. Compared to previous research findings however (Kuntz et al., 2015), our modelling casts doubt on the substantive importance of a country’s legal and policy framework regards homosexuality in either affecting homopositive attitude directly or in moderating the effects of basic human values on homopositivity.

Taken together the analyses add a range of valuable new insights to our understanding of the key predictors, pathways and moderators of homopositive attitudes. Despite general strides towards greater acceptance of homosexuality homonegativity continues to be a challenge in many regions of the world and within certain demographic groups of all nations. It is our hope that this development in the literature of these richer understandings of homopositive attitudes beyond the identification of headline predictors can help to support continued progress towards equality and acceptance of homosexuality across all people and places.

## Data Availability

The ESS data are available publicly to download at: https://www.europeansocialsurvey.org/data/

## Footnotes

1 “low” = lower secondary and below; “medium” = upper secondary; “high” = advanced vocational and tertiary.

2 “good health” = values 1 and 2 on the underlying health variable; “poor health” = values 3 to 5 on the underlying health variable.

3 “poor life satisfaction” = values 0 to 6 on the underlying variable; “high life satisfaction” = values 7 to 10 on the underlying variable.

## Code Availability

The full Stata code is available on request from the corresponding author.
